# Supplementary material for: Heat adaptation of phage T7 under an extended genetic code
Source: Virus Evol. 2021 Dec 1;7(2):veab100. doi: 10.1093/ve/veab100 (PMC8923235; doi:10.1093/ve/veab100)
Supplement: veab100_Supp [file veab100_supp.zip › Sequencing_Analysis.zip!/Sequencing_Analysis/S1/evidence/SNP_4.html]

BRESEQ :: Evidence


| Predicted mutation | | | | | | |
| --- | --- | --- | --- | --- | --- | --- |
| evidence | seq id | position | mutation | annotation | gene | description |
| RA | NC\_001604.1 | 18,406 | G→A | intergenic (–/–) | – / – | –/– |

| Read alignment evidence... | | | | | | | | | | | |
| --- | --- | --- | --- | --- | --- | --- | --- | --- | --- | --- | --- |
|  | seq id | position | | ref | new | freq | score (cons/poly) | reads | annotation | genes | product |
| \* | NC\_001604.1 | 18,406 | 0 | G | A | 99.7% | 12541.3 / ‑3.0 | 4839 | intergenic (–/–) | *–/–* | –/– |
| Reads supporting (aligned to +/- strand):  **ref** base G (5/9);  **new** base A (2506/2295);  **total** (2526/2313) | | | | | | | | | | | |
| Fisher's exact test for biased strand distribution *p*-value = 2.86e-01 | | | | | | | | | | | |
| Kolmogorov-Smirnov test that lower quality scores support variant *p*-value = 9.96e-01 | | | | | | | | | | | |

Only 100 of 4840 total aligned reads displayed.  

`ATGGTTACTCAGGGATTGCTGGATGGGGTGATACCGCCGAGGACTTCTTGAATAACCCGTTCATAACCGAGCCTAAAACGTCTGTGCTTAAGTCCGGTAAGAACAAAGGCCAAGAGGTTACTAAATGGGTTAAACGCGACCCTGAGCCTCATGAGACGCTTTGGGACTGCATTAAGTCCATTGGCGCGAAGGCTGGTATGACCGAAGAGGATATTATCAAGCAGGGCCAAATGGCTCGAATCCTACGGTTCAACGAGTACAACTTTATTGACAAGGAGATTTACCTGTGGAGACCGTAGCGTATATTGGTCTGGGTCTTTGTGTTCTCGGA‑‑GTGTGCCTCATTTCGTGGGGCCTTTGGGACTTAGCCAGAATAATCAAGTCGTTACACGACACTAAGTGATAAACTCAAGGTCCCTAAATTAATACGACTCACTATAGGGAGATAGGGGCCTTTACGATTATTACTTTAAGATTTAACTCTAAGAGGAATCTTTATTATGTTAACACCTATTAACCAATTACTTAAGAACCCTAACGATATTCCAGATGTACCTCGTGCAACCGCTGAGTATCTACAGGTTCGATTCAACTATGCGTA  >  NC_001604.1/18108‑18705`  

                                                                                                                                                                                                                                                                                                          `|`                                                                                                                                                                                                                                                                                                               
 `tGGTTACTCAGGGATTGCTGGATGGGGTGATACCGCCGATGACTTCTTGAATAACCCGTTCATAACCGAGCCTAAAACGTCTGTGCTTAAGTCCGGTAAGAACAAAGGCCAAGAGGTTACTAAATGGGTTAAACGCGACCCTGAGCCTCATGAGACGCTTTGGGACTGCATTAAGTCCATTGGCGCGAAGGCTGGTATGACCGAAGAGGATATTATCAAGCAGGGCCAAATGGCTCGAATCCTACGGTTCAACGAGTACAACTTTATTGACAAGGAGATTTACCTGTGGAGACCGTAACGt                                                                                                                                                                                                                                                                                                            >  1:44462/1‑301 (MQ=255)`  
 `tGGTTACTCAGGGATTGCTGGATGGGGTGATAACGCCGAGGACTTCTTGAATAACCCGTTCATAACCGAGCCTAAAACGTCTGTGCTTAAGTCCGGTAAGAACAAAGGCCAAGAGGTTACTAAATGGGTTAAACGCGACCCTGAGCCTCATGAGACGCTTTGGGACTGCATTAAGTCCATTGGCGCGAAGGCTGGTATGACCGAAGAGGATATTATCAAGCAGGGCCAAATGGCTCGAATCCTACGGTTCAACGAGTACAACTTTATTGACAAGGAGATTTACCTGTGGAGACCGTAACGt                                                                                                                                                                                                                                                                                                            <  1:58666/301‑1 (MQ=255)`  
   `gTTACTCAGGGATTGCTGGATGGGGTGATACCGCCGAGGACTTCTTGAATAACCCGTTCATAACCGAGCCTAAAACGTCTGTGCTTAAGTCCGGTAAGAACAAAGGCCAAGAGGTTACTAAATGGGTTAAACGCGACCCTGAGCCTCATGAGACGCTTTGGGACTGCATTAAGTCCATTGGCGCGAAGGCTGGTATGACCGAAGAGGATATTATCAAGCAGGGCCAAATGGCTCGAATCCTACGGTTCAACGAGTACAACTTTATTGACAAGGAGATTTACCTGTGGAGACCGTAACGtat                                                                                                                                                                                                                                                                                                          <  1:344396/301‑1 (MQ=255)`  
        `tCAGGGATTGCTGGAAGGGGTGATACCGCCGAGGACTTCTTGAATAACCCGTTCATAACCGAGCCTAAAACGTCTGTGCTTAAGTCCGGTAAGAACAAAGGCCAAGAGGTTACTAAATGGGTTAAACGCGACCCTGAGCCTCATGAGACGCTTTGGGACTGCATTAAGTCCATTGGCGCGAAGGCTGGTATGACCGAAGAGGATATTATCAAGCAGGGCCAAATGGCTCGAATCCTACGGTTCAACGAGTACAACTTTATTGACAAGGAGATTTACCTGTGGAGACCGTAACGTATATTgg                                                                                                                                                                                                                                                                                                     <  1:24004/301‑1 (MQ=255)`  
        `gcAGGGATTGCTGGATGGGGTTATACCGCCGAGGACTTCGTGAATAACCCGTTAATAACCGAGCCTAAAACGTCTGTGCTTAAGTCCGGTAAGAACAAAGGCCAAGTGGTTACTAAATGGGTTAAACGCGACCCTGAGCCTCATGAGACGCTTTGGGACTGCATTAAGTCCATTGGCGCGAAGGCTGGTATGACCGAAGAGGATATTATCAAGCAGGGCCAAATGGCTCGAATCCTACGGTTCAACGAGTACAACTTTATTGACAAGGAGATTTACCTGTGGAGACCGTAACGTATATTgg                                                                                                                                                                                                                                                                                                     <  1:663479/300‑1 (MQ=255)`  
          `aGGGATTGCTGGATGGGGTGATACCGCCGAGGACGTCTTGAATAACCCGTTCATAACCGAGCCTAAAACGTCTGTGCTTAAGTCCGGTAAGAACAAAGGCCAAGAGGTTACTAAATGGGTTAAACGCGACCCTGAGCCTCATGAGACGCTTTGGGACTGCATTAAGTCCATTGGCGCGAAGGCTGGTATGACCGAAGAGGATATTATCAAGCAGGGCCAAATGGCTCGAATCCTACGGTTCAACGAGTACAACTTTATTGACAAGGAGATTTACCTGTGGAGACCGTAACGTATACTGGTc                                                                                                                                                                                                                                                                                                   <  1:496787/301‑1 (MQ=255)`  
           `gggATTGCTGGATGGGGTGATACCGCCGAGGACTTCTTGAATAACCCGTTCATAACCGAGCCTAAAACGTCTGTGCTTAAGTCCGGTAAGAACAAAGGCCAAGAGGTTACTAAATGGGTTAAACGCGACCCTGAGCCTCATGAGACGCTTTGGGACTGCATTAAGTCCATTGGCGCGAAGGCTGGTATGACCGAAGAGGATATTATCAAGCAGGGCCAAATGGCTCGAATCCTACGGTTCAACGAGTACAACTTTATTGACAAGGAGATTTACCTGTGGAGACCGTAACGTATATTGGTCt                                                                                                                                                                                                                                                                                                  >  1:455654/1‑301 (MQ=255)`  
                 `gCTGGATGGGGTGATACCGCCGAGGACTTCTTTAATAACCCGTTCATAACCGAGCCTAAAACGTCTGTTCTTAAGTCCGGTAAGAACAAAGGCCAAGAGGTTACTAAATGGGTTAAACGCGACCCTGAGCCTCATGAGACGCTTTGGGACTGCATTAAGTCCATTGGCGCGAAGGCTGGTATGACCGAAGAGGATATTATCAAGCAGGGCCAAATGGCTCGAATCCTACGGTTCAACGAGTACAACTTTATTGACAAGGAGATTTACCTGTGGAGACCGTAACGTATATTGGTCTGGGTCt                                                                                                                                                                                                                                                                                            >  1:134168/1‑301 (MQ=255)`  
                 `gCTGGATGGGGTGATACCGCCGAGGACTTCTTGAATAACCCGTTCATAACCGAGCCTAAAACGTCTGTGCTTAAGTCCGGTAAGAACAAAGGCCAAGAGGTTACTAAATGGGTTAAACGCGACCCTGAGCCTCATGAGACGCTTTGGGACTGCATTAAGTCCATTGGCGCGAAGGCTGGTATGACCGAAGAGGATATTATCAAGCAGGGCCAAATGGCTCGAATCCTACGGTTCAACGAGTACAACTTTATTGACAAGGAGATTTACCTGTGGAGACCGTAACGTATATTGGTcctgtctc                                                                                                                                                                                                                                                                                            >  1:273127/1‑294 (MQ=255)`  
                    `ggATTGGGTGATACCGCCGAGGACTTCTTGAATAACCCGTTCATAACCGAGCCTAAAAAGTTGGTGCTTAAGGCCGGTAAAGACAAAGGCCAAGAGGTTACTAAATGGGTTAAACGCGACCCTGAGCCTCATGAGACGCTTTGGGACTGCATTAAGTCCATTGGCGCAAAGGCTGGTATGACCGAAGAGGATATTATCAAGCAGGGCCAAATGGCTCGAATCCTACGGTTCAACGAGTACAACTTTATTGACAAGGAGATTTACCTGTGGAGACCGTAACGTATATTGGTCTGGGTCTTtg                                                                                                                                                                                                                                                                                         <  1:52964/301‑1 (MQ=255)`  
                    `ggATGGGGTGATACCGCCGAGGACTTCTTGAATAACCCGTTCATAACCGAGCCTAAAACGTCTGTGCTTAAGTCCGGTAAGAACAAAGGCCAAGAGGTTACTAAATGGGTTAAACGCGACACTGAGCCTCATGAGACGCTTTGGGACTGCATTAAGTCCATTGGCGCGAAGGCTGGTATGACCGAAGAGGATATTATCAAGCAGGGCCAAATGGCTCGAATCCTACGGTTCAACGAGTACAACTTTATTGACAAGGAGATTTACCTGTGGAGACCGTAACGTATAttctgtctcttatacc                                                                                                                                                                                                                                                                                         >  1:234531/1‑287 (MQ=255)`  
                         `gggTGATACCGCCGAGGACTTCTTGAATAACCCGTTCATAACCGAGCCTAAAACGTCTGTGCTTAAGTCCGGTAAGAACAAAGGCCAAGAGGTTACTAAATGGGTTAAACGCGACCCTGAGCCTCATGAGACGCTTTGGGACTGCATTAAGTCCATTGGCGCGAAGGCTGGTATGACCGAAGAGGATATTATCAAGCAGGGCCAAATGGCTCGAATCCTACGGTTCAACGAGTACAACTTTATTGACAAGGAGATTTACCTGTGGAGACCGTAACGTATATTGGTCTGGGTCTTTGTGttt                                                                                                                                                                                                                                                                                    >  1:128603/1‑300 (MQ=255)`  
                                 `ccgccgAGGACTTATTGAATAACCCGTTCAAAACCGAGCCTAAAACGTCTGTGCTTAAGTCCGGTAAGAACAAAGGCCAAGAGGTTACTAAATGGGTTAAACGCGACCCTGAGCCTCATGAGACGCTTTGGGACTGCATTAAGTCCATTGGCGCGAAGGCTGGTATGACCGAAGAGGATATTATCAAGCAGGGCCAAATGGCTCGAATCCTACGGTTCAACGAGTACAACTTTATTGACAAGGAGATTTACCTGTGGAGACCGTAACGTATATTGGTCTGGGTCTTTGTGTTCTCGGA‑‑gtg                                                                                                                                                                                                                                                                          <  1:562979/301‑1 (MQ=255)`  
                                  `cgcgtaGGACTTCTTGAATAACCCGTTCATAACCGAGCATACAACGTCTGTGCTTAAGGCCGGTAAGAACAAAGGCCAAGAGGTTACTAAATGGGTTAAACGCGACCCTGAGCCTAATGAGACGCTTTGGGACTGCATTAAGTCCATTGGCGCGAAGGCTGGTATGACCGAAGAGGATATTATCAAGCAGGGCCAAATGGCTCTAATCCTACGGTTCAACGAGTACAACTTTATTGACAAGGAGATTTACCTGTGGAGACCGTAACGTATATTGGTCTGGGTCTTTGTGTTCTCGGA‑‑gtgt                                                                                                                                                                                                                                                                         <  1:81111/296‑1 (MQ=255)`  
                                        `ggACTTCTTGAATAACCCGTTCATAACCTAGCCTAAAACGTCTGTGCTTAAGTCCGGTAAGAACAAAGGCCAAGAGGTTACTAAATGGGTTAAACGCGACCCTGAGCCTCATGAGACGCTTTGGGACTGCATTAAGTCCATTGGCGCGAAGGCTGGTATGACCGAAGAGGATATTATCAAGCAGGGCCAAATGGCTCGAATCCTACGGTTCAACGAGTACAACTTTATTGACAAGGAGATTTACCTGTGGAGACCGTAACGTATATTGGTCTGGGTCTTTGTGTTCTCGGA‑‑GTGTGCCTCa                                                                                                                                                                                                                                                                   >  1:592463/1‑301 (MQ=255)`  
                                         `aaCTTCTTAAATAACCCGTTCATAACCGAGCCTAAAACGTGTGTGATTAAGTCCGGTAAGAACAAAGGCCAAGAGGTTACTAAATGGGTTAAACGCGACCCTGAGCATCATGAGACGCTTTGGGACTGCATTAAGTCCATTGGCGCGAAGGCTGGTATGACCGAAGAGGATATTATCAAGCAGGGCCAAATGGCTCGAATCCTGCGGTTCAACGAGTACAACTTTATTGACAAGGAGATTTACCTGTGGAGACCGTAACGTATATTGGTATGGGTCTTTGTGTTCTCGGA‑‑GTGTGCCTCAt                                                                                                                                                                                                                                                                  <  1:121052/300‑1 (MQ=255)`  
                                           `cttcttGAATAACCCGTTCATAACCGGGCCTAAAACGTCTGTGCTTAAGTCCGGTAAGAACAAAGGCCAAGAGGTTACTAAATGGGTTAAACGCGACCCTGAGCCTCATGAGACGCTTTGGGACTGCATTAAGTCCATTGGCGCGAAGGCTGGTATGACCGAAGAGGATATTATCAAGCAGGGCCAAATGGCTCGAATCCTACGGTTCAACGAGTACAACTTTATTGACAAGGAGATTTACCTGTGGAGACCGTAACGTATATTGGTCTGGGTCTTTGTGTTCTCGGA‑‑GTGTGCCTCAttt                                                                                                                                                                                                                                                                <  1:428962/301‑1 (MQ=255)`  
                                            `ttcttGAATAACCCGTTCATAACCGAGCCTAAAACGTCTGTGCTTAAGTCCGGTAAGAACAAAGGCCAAGAGGTTACTAAATGGGTTAAACGCTACCCTGAGCCTCATGAGACGCTTTGGGACTGCATTAAGTCCATTGGCGCGAAGGCCGGTATGACCGAAGAGGATATTATCAAGCAGGGTCAAATGGCTCGAATCCTACGGTTCAACGAGTACAACTTTATTGACAAGGAGATTTACCTGTGGAGACCGTAACGTATATTGGTCTGGGTCTTTGTGTTCTCGGA‑‑GTGTGCCTCATTTc                                                                                                                                                                                                                                                               >  1:93968/1‑301 (MQ=255)`  
                                                `tGAATAACCCGTTCATAACCGAGCCTAAAACCTCTGTGCTTAAGTCCGGTAAGAACAAAGGCCAAGAGGTTACTAAATGGGTTAAACGCGACCCTGAGCCTCATGAGACGCTTTGGGACTGCATTAAGTCCATTGGCGCGAAGGCTGGAATGACCAAAGAGGATATTATCAAGCAGGGCCAAATGGCTCGAATCCTACGGTTCAACGAGTACAACTATATTAACAAGAGGATTTACCTGTGGAGACCGTAACGAAAATTGGTCTGGGTCTTTGTGTTCTCAGA‑‑GTGTGCCTAATTTCGTgg                                                                                                                                                                                                                                                           >  1:490474/1‑301 (MQ=255)`  
                                                    `aaaCCCGTTCATAACCGAGCCTAAAACGTCTGTGCTTAAGTCCGGTAAGAACAAAGGCCAAGAGGTTACTAAATGGGTTAAACGCGACCCTGAGCCTCATGAGACGCTTTGGGACTGCATTAAGTCCATTGGCGCGAAGGCTGGTATGACCGAAGAGGATATTATCAAGCAGGGCCAAATGGCTCGAATCCTACGGTTCAACGAGTACAACTTTATTGACAAGGAGATTTACCTGTGGAGACCGTAACGTATATTGGTCTGGGTCTTTGTGTTCTCGGA‑‑GTGTGCCTCATTTCGTGGGGcc                                                                                                                                                                                                                                                       <  1:581839/300‑1 (MQ=255)`  
                                                          `tttCATAACCGAGCCTAAAACGTCTGTGCTTAAGTCCGGTAAGAACAAAGGCCAAGAGGTTACTAAATGGGTTAAACGCGACCCTGAGCCTCATGAGACGCTTTGGGACTGCATTAAGTCCATTGGCGCGAAGGCTGGTATGACCGAAGAGGATATTATCAAGCAGGGCCAAATGGCTCGAATCCTACGGTTCAACGAGTACAACTTTATTGACAAGGAGATTTACCTGTGGAGACCGTAACGTATATTGGTCTGGGTCTTTGTGTTCTCGGA‑‑GTGTGCCTCATTTCGTGGGGCCTTTggg                                                                                                                                                                                                                                                 <  1:321322/300‑1 (MQ=255)`  
                                                          `gTTCATAACCGAGCCTAAAACGTCTGTGCTCAAGTCCGGTAAGAACAAAGGCCAAGAGGTTACTAAATGGGTTAAACGCGACCCTGAGCCTCATGAGACGCTTTGGGACTGCATTAAGTCCATTGGCGCGAAGGCTGGTAGGACCGAAGAGGATATTATCAAGCAGGGCCAAATGGCTCGAATCCTACGGTTCAACGAGTACAACTTTATTGACAAGGAGATTTACCTGTGGAGACCGTAACGTATATTGGAATGGGTCTTTGTGTTCTCGGA‑‑GTGTGCCTCATTTCGTGGGTCGTTgggg                                                                                                                                                                                                                                                 >  1:612524/1‑301 (MQ=255)`  
                                                            `ataagagacaGCCTAAAACGTGTGTGCTTAAGTCCGGTAAGAACAAAGGCCAAGAGGTTACTAAATGGGTTAAACGCGACCCTGAGCCTCATGAGACGCTTTGGGACTGCATTAAGTCCATTGGCGCGAAGGCTGGTATGACCGAAGAGGATATTATCAAGCAGGGCCAAATGGCTCGAATCCTACGGTTCAACGAGTACAACTTTATTGACAAGGAGATTTACCTGTGGAGACCGTAACGTATATTGGTCTGGGTCTTTGTGTTCTCGGA‑‑GTGTGCCTCATTTCGTGGGGCCTTTGGGAc                                                                                                                                                                                                                                               <  1:160046/292‑1 (MQ=255)`  
                                                                              `cGTCTGTGCTTAAGTCCGGTAAGAACAAAGGCCAAGAGGTTACTAAATGGGTTAAACGCGACCCTGAGCCTCATGAGACGCTTTGGGACTGCATTAAGTCCATTGGCGCGAAGGCTGGTATGACCGAAGAGGATATTATCAAGCAGGGCCAAATGGCTCGAATCCTACGGTTCAACGAGTACAACTTTATTGACAAGGAGATTTACCTGTGGAGACCGTAACGTATATTGGTCTGGGTCTTTGTGTTCTCGGA‑‑GTGTGCCTCATTTCGTGGGGCCTTTGGGACTTAGCCAGAATAATCaat                                                                                                                                                                                                                             >  1:277241/1‑300 (MQ=255)`  
                                                                                     `gCTTAAGTCCGGTAAGAACAAAGGCCAAGAGGTTACTAAATGGGTTAAACGCGACCCTGAGCCTCATGAGACGCTTTGGGACTGCATTAAGTCCATTGGCGCGAAGGCTGGTATGACCGAAGAGGATATTATCAAGCAGGGCCAAATGGCTCGAATCCTTCGGTTCAACGAGTACAACTGTATTGACAAGGAGATTTACCTTTGGAGACCGTAACGTATATTGGTCCTGGGCTTTGTGCTCTCGGA‑‑GTGTGCCTCATTTCGTGGGGCCTTTGGGCCGTAGCCAGAATAATCAAGTCGTTaa                                                                                                                                                                                                                      >  1:221370/1‑300 (MQ=255)`  
                                                                                          `aGTCCGGTAAGAACAAAGGCCAAGAGGTTACTAAATGGGTTAAACGCGACCATGAGCCTCATGAGACGCTTTGGGACTGCATTAAGTCCATTGGCGCGAAGGCTGGTATGACCGAAGAGGATATTATCAAGCAGGGCCAAATGGCTCGAATCCTACGGTTCAACGAGTACAACTTTATTGACAAGGAGATTTACCTGTGGAGACCGTAACGTATATTGGTCTGGGTCTTTGTGTTCTCGGA‑‑GTGTGCCTCATTTCGTGGGGCCTTTGGGACTTAGCCAGAATAATCAAGTCGTTACACGac                                                                                                                                                                                                                 <  1:468358/301‑1 (MQ=255)`  
                                                                                                `gTAAGAACAAAGGCCAAGAGGTTACTAAATGGGTTAAACGCGACCCTGAGTCTCATGGGACGCGTGGGGACTGCATTCAGTCCATTGGCGCGAAGGCTGGTATGACCGAAGAGGATATTATCAAGCAGGGCCAAATGGCTCGAATCCTACGGTTAAACGAGTACAACTTTATTGACAGGGAGATTTATCTGTGGAGACCGTAACGTATATGGGTCTGGGTCTTTGTGTTCTCGGA‑‑GTGTGCCTCATTTCGTGGGGCCTTTGGGACTTAGCCAGAATAATCAAGTCGTTACACGACACTAAg                                                                                                                                                                                                           <  1:47956/301‑1 (MQ=255)`  
                                                                                                    `gAACAAAGGCCAAGAGGTTACTAAATGGGTTAAACGCGACCCTGAGCCTCATGAGACGCTTTGGGACTGCATTAAGTCCATTGGCGCGAAGGCTGGTATGACCGAAGAGGATATTATCAAGCAGGGCCAAATGGCTCGAATCCTACGGTTCAACGAGTACAACTTTATTGACAAGGAGATTTACCTGTGGAGACCGTAACGTATATTGGTCTGGGTCTTTGTGTTCTCGGA‑‑GTGTGCCTCATTTCGTGGGGCCTTTTGGACTTAGCCAGAATAATCAAGTCGTTACACGACACTACGttct                                                                                                                                                                                                       >  1:410901/1‑298 (MQ=255)`  
                                                                                                                    `gTTACTAAATGGGTTAAACGCGACCCTGAGCCTCATGAGACGCTTTGGGACTGCATTAAGTCCATTGGCGCGAAGGCTGGTATGACCGAAGAGGATATTATCAAGCAGGGCCAAATGGCTCGAATCCTACGGTTCAACGAGTACAACTTTATTGACAAGGAGATTTACCTGTGGAGACCGTAACGTATATTGGTCTGGGTCTTTGTGTTCTCGGA‑‑GTGTGCCTCATTTCGTGGGGCCTTTGGGACTTAGCCAGAATAATCAAGTCGTTACACGACACTAAGTGATAAACTCAAGGTCCCTa                                                                                                                                                                                       >  1:153560/1‑301 (MQ=255)`  
                                                                                                                       `aCTAAATGGGTTAAACGCGACCCTAAGCCTCATGAGACGCTTTGGGACTGCATTAAGTCCATTGGCGCGAAGGCTGGTATGACCGAAGAGGATATTATCAAGCAGGGCCAAATGGCTCGAATCCTACGGTTCAACGAGTACAACTTTATTGACAAGGAGATTTACCTGTGGAGACCGTAACGTATATTGGTCTGGGTCTTTGTGTTCTCGGA‑‑GTGTGCCTCATTTCGTGGGGCCTTTGGGACTTAGCCAGAATAATCAAGTCGTTACACGACACTAAGTGATAAACTCAAGGTCCCTAAAt                                                                                                                                                                                    >  1:326673/1‑301 (MQ=255)`  
                                                                                                                        `cTAAATGGGTTAAACGCGACCCTGAGCCTCATGAGACGCTTTGGGACTGCATTAAGTCCATTGGCGCGAAGGCTGGTATGACCGAAGAGGATATTATCAAGCAGGGCCAAATGGCTCGAATCCTACGGTTCAACGAGTACAACTTTATTGACAAGGAGATTTACCTGTGGAGACCGTAACGTATATTGGTCTGGGTCTTTGTGTTCTCGGA‑‑GTGTGCCTCATTTCGTGGGGCCTTTGGGACTTATCCAGAATAATCAAGTCGTTACACGACACTAAGTGATAAACTCAAGGTCCCTAAAtt                                                                                                                                                                                   <  1:147218/301‑1 (MQ=255)`  
                                                                                                                            `acggggttaacggtaccccgtacatCATGGGACGATTTGGTACGGCATGAAGTCCATTGGCGCGAAGGCTGGTATTACCGAATAGGAGATTATCAAGCAGGGACAAATCGCTCGAATACTTCTGTTCAACGCGTACCACTTTATTGACAAGGATATTTACCTGTGGAGACCGTAACGTTTATTGGTCTGGGTCTTTGTGTTCTCGGA‑‑GTGTGCCTCATTTCGTGGGGCCTTTAGGACTTAGCCATAATAATCAAGTCGTTACACGACACTACGTGATAAACTCAAGGTCCCTAAATTAATa                                                                                                                                                                               <  1:372782/277‑1 (MQ=255)`  
                                                                                                                             `tggcgtAAACGCGACCCTGAGCCTAATGAGACGTTGTGGGACTGCATTAAGTCCATTGGCGCGAAGGCTGGGATGACGGAAGAGGATATTATCAAGCAGTGCCAAATGGCTCGAATCCTACGGTTCAACGAGTACAACTTGATTGACAAGGAGATTTACCTGTGGAGACCGTAACGTATATTGGTCTGGGTCTTTGTGTTCTCGGA‑‑GTGTGCCTCATTTCGTGGGGCCTTTGGGACTTAGCCAGAATAATCAAGTCGTTACACGACACTAAGTGATAAACTCAAGGTCCCTAAATTAATAc                                                                                                                                                                              <  1:114793/296‑1 (MQ=255)`  
                                                                                                                               `ggTTAAACGCGACCCTGAGCCTCATGAGACGCTTTGGGACTGCATTAAGTCCATTGGCGCGAATGCTGGTATGACCGAAGAGGATATTATCAAGCAGGGCCAAATGGCTCGAATCCTACGGTTCAACGAGTACAACTTTATTGACAAGGAGATTTACCTGTGGAGACCGTAACGTATATTGGTCTGGGTCTTTGTGTTCTCGGA‑‑GTGTGCCTCATTTCGTGGGGCCTTTGGGACTTAGCCAGAATAATCAAGTCGTTACACGACACTAAGTGATAAACTCAAGGTCCCTAAATTCATACGa                                                                                                                                                                            >  1:70515/1‑301 (MQ=255)`  
                                                                                                                                       `gcgACCCTGAGCCTCATGAGACGCTTTGGGACTGAATTAAGGCCATTGGCGCGAAGGCTGGTATGACCGAAAAGGATATTATCAAGCAGGGCCAAATGGCTCGAATCCTACGGTTCAACGAGTACAACTTTATTGACAAGGAGATTTACCTGTGGAGACCGTAACGTATATTGGTCTGGGTCTTTGTGTTCTCGGA‑‑GTGTGCCTCATTTCGTGGGGCCTTTGGGACTTAGCCAGAATAATCAAGTCGTTACACGACACTAAGTGATAAACTCAAGGTCCCTAAATTAATACGACTCACtat                                                                                                                                                                    <  1:139635/301‑1 (MQ=255)`  
                                                                                                                                          `aCCCTGAGCCTCATGAGACGCTTTTGGACTGCATTAAGTCCATTGGCGCGAAGGCTGGTATGACCGAAGAGGATATTATCAAGCAGGGCCAAATGGCTCGAATCCTACGGTTCAACGAGTACAACTTTATTGACAAGGAGATTTACCTGTGGAGACCGTAACGTATATTGGTCTGGGTCTTTGTGTTCTCGGA‑‑GTGTGCCTCATTTCGTGGGGCCTTTGGGACTTAGCCAGAATAATCAAGTCGTTACACGACACTAAGTGATAAACTCAAGGTCCCTTAATTAATACGACTCACTATAgg                                                                                                                                                                 >  1:546320/1‑301 (MQ=255)`  
                                                                                                                                          `aCCCTGAGCCTCATGAGACGCTTTGGGACTGCATTAAGTCCATTGGCGCGAAGGCTGGTATTACCGAAGAGGATATTATCAAGCAGGGCCAAATGGCTCGAATCCTACGGTTCAACGAGTACAACTTTATTGACAAGGAGATTTACCTGTGGTGACAGTAACTTATATCGGTCTGGGTCTTTGTGTTCTCTGA‑‑ATGTGGCTCATTTCGTGGGGGCTTTGGGACTTAGCCAGGATAAACAAGTCGTTACAAGAGACTAAGTGATAAACTCAAGTTCCCT‑ACTTAAAACGACTCACTATAggg                                                                                                                                                                >  1:616921/1‑301 (MQ=255)`  
                                                                                                                                          `aCCCTGAGCCTCATGAGACGCTTTGGGACTGCATTAAGTCCATTGGCGCGAAGGCTGGTATGACCGAAGAGGATATTATCAAGCAGGGCCAAATGGCTCGAATCCTACGGTTCAACGAGTACAACTTTATTGACAAGGAGATTTACCTGTGGAGACCGTAACGTATATTGGTCTGGGTCTTTGTGTTCTCGGA‑‑GTTTGCCCCATTTCGTGGGGCCTTTGGGACTTAGCCAGAATAATCAAGTATTTACACGACACTAAGTGATAAACTCAAGGTCCCTAAATTAATACGACTCACACTAgg                                                                                                                                                                 >  1:225818/1‑301 (MQ=255)`  
                                                                                                                                          `aCCCTGAGCCTCATGAGACGCTTTGGGACTGCATTAAGTCCATTGGCGCGAAGGCTGGTATGACCGAAGAGGATATTATCAAGCAGGGCCAAATGGCTCGAATCCTACGGTTCAACGAGTACAACTTTATTGACAAGGAGATTTACCTGTGGAGACCGTAACGTATATTGGTCTGGGTCTTTGTGTTCTCGGA‑‑GTGTGCCTCATTTCGTGGGGCCTTTGGGACTTAGCCAGAATAATCAAGTCGTTACACGACACTAAGTGATAAACTCAAGGTCCCTAAATTAATACGACTCACTATAgg                                                                                                                                                                 >  1:574855/1‑301 (MQ=255)`  
                                                                                                                                           `cccTGAGCCTCATGGGACGCTTTGGGACTGCATTAAGTCCATTGGCGCGAAGGCTGGTATGACCGAAGAGGATATTATCAAGCAGGTCCAAATGGCTCGAATCCTACGGTTCAACGAGTACAACTTTATTGACAAGGAGATTTACCTGTGGAGACCGTAACGTATATTGGTCTGGGTCTTTGTGTTCGCGGA‑‑GTGTGCCTCATTTCGTGGGGCCTTTGGGACTTAGCCAGAAGAATCAAGTCGTTACACGACACTAAGTGATAAACTCAAGGTCCCTAAATTAATACGACTCACTATAggg                                                                                                                                                                <  1:310707/301‑1 (MQ=255)`  
                                                                                                                                            `gatgtgtataagagacatcgtagggACTGCATTAAGGCCATTGGCGCGAAGGCTGGTATGACCGAAGAAGATATTATCAAGCAGGGCCAAATGGCTCGAATCCGACGGTTCAACGAGTACAACTTTATTGACAAGGAGATTTACCTGTGGAGACCGTAACGTATATTGGTCTGGGTCTTTGTGTTCTCGGA‑‑GTGTGCCTCATTTCGTGGGGCCTTTGGGACTTAGCCAGAATAATCAAGTCGTTACACGACACTAAGTGATAAACTCAAGGTCCCTAAATTAATACGACTCACTATAGGga                                                                                                                                                               <  1:473851/279‑1 (MQ=255)`  
                                                                                                                                            `ccTGGGCCTCATGAGACGCTTTGGGACTGCATTAAGTCCATTGGCGCGAAGGCTGGTATGACCGAAGAGGATATTATCAAGCAGGGCCAAATGGCTCGAATCCTACGGTTCAACGAGTACAACTTTATTGACAAGGAGATTTACCTGTGGAGACCGTAACGTATATTGGTCTGGGTCTTTGTGTTCTCGGA‑‑GTGTGCCTCATTTCGTGGGGCCTTTGGGACTTAGCCAGAATAATCAAGTCGTTACACGACACTAAGTGATAAACTCAAGGTCCCTAAATTAATACGACTCACTATAGGga                                                                                                                                                               <  1:434165/301‑1 (MQ=255)`  
                                                                                                                                             `acgacagtCATGGGACGCTTTGGTATTGCAGTACTGCCATTAGCGCGAAGGCTGGGATGACCGAAGAGGATATTATAAAGCAGGGCCAAATGGCTCGAATCCTACGGTTCAACGAGTACAACTTTATTGACAAGGAGATTTACCTGTGGAGACCGTA‑CGTATATTGGTCTGGGTCTTTGTGTTCTCGGAGTGTGTGCCTCATTTCGTGGGGCCTTTGGGACTTAGCCAGAATAATCAAGTCGTTACACGACACTAAGTGATAAACTCAAGGTCCCTAAATTAATACGACTCACTATAGGga                                                                                                                                                               <  1:630693/294‑1 (MQ=255)`  
                                                                                                                                              `tGAGCCTCAGGAGACGCTTTGGGACTGCATTAAGTCCATTGGCGCGAAGGCTGGTATGACCGAAGAGGATATTATCAAGCAGGGCCAAATGGCTCGAATCCTACGGTTCAACGAGTACAACTTTATTGACAAGGAGATTTACCTGTGGAGACCGTAACGTATATTGGTCTGGGTCTTTGTGTTCTCGGA‑‑GTGTGCCTCATTTCGTGGGGCCTTTGGGACTTAGCCAGAATAATCAAGTCGTTACACGACACTAAGTGATAAACTCAAGGTCCCTAAATTAATACGACTCACTATAGGgaga                                                                                                                                                             <  1:34999/301‑1 (MQ=255)`  
                                                                                                                                                 `gCCTCATGAGACGCTTTGGGACTGCATTAAGTCCATTGGCGCGAAGGCTGGTATGACCGAAGAGGATATTATCAAGCAGGGCCAAATGGCTCGAATCCTACGGTTCAACGAGTACAACTTTATTGACAAGGAGATTTACCTGTGGAGACCGTAACGTATATTGGTCTTGGTCTTTGTGTTCTCGGA‑‑GTGTGCCTCATTTCGAGGGGCCTTTGGGACTTAGCCAGAATAATCAAGTCGTTACACGACACTAAGTGATAAACTCAAGGTCCCTAAATTAATACGACTCACTATAGGgagactg                                                                                                                                                          >  1:485324/1‑298 (MQ=255)`  
                                                                                                                                                  `ccTCATGAGACGCTTTGGGACTGCATTAAGTCCATTGGCGCGAAGGCTGGTATGACCGAAGAGGATATTAGCAAGCAGGGCCAAATGGCTCGAATCCTACGGTTCAACGAGTACAACTTTATTGACAAGGAGATTTACCTGTGGAGACCGTAACGTATAGTGGTCTGGGTCTTTGTGTTCTCGGA‑‑GTGTGCCTCATTTCGTGGGGCCTTTGGGACTTAGCCAGAATAATCAAGTCGTTACACGACACTAAGTGATAAACTCAAGGCCCCTAAATTAATACGACTCACTATAGGGAGATAgg                                                                                                                                                         <  1:252761/301‑1 (MQ=255)`  
                                                                                                                                                   `cTCATGAGACGCTTTGGGACTGCATTAAGTCCATTGGCGCGAAGGCTGGTATGACCGAAGAGGATATTATCAAGCAGGGCCAAATGGCTCGAATCCTACGGTTCAACGAGTACAACTTTATTGACAAGGAGATATACCTGTGGAGACCGTAACGTATATTGGTCTGGGTCTTTGTGTTCTCGGA‑‑GTGTGCCTCATTTCGTGGGGCCTTTGGGACTTAGCCAGAATAATCAAGTCGTTACACGACACTAAGTGATAAACTCAAGGTCCCTAAATTAATACGACTCACTATAGGGAGATAggg                                                                                                                                                        <  1:534582/301‑1 (MQ=255)`  
                                                                                                                                                     `cATTAGACGCTTTGGGACTGCATTAAGTCCATTGGCGCGAAGGCTGGTATGACCGAAGAGGATATTATCAAGCAGGGCCAAATGGCTCGAATCCTACGGTTCAACGAGTACAACTTTATTGACAAGGAGATTTACCTGTGGAGACCGTAACGTATATTGGTCTGGGTCTTTGTGTTCTCGGA‑‑GTGTGCCTCATTTCGTGGGGCCTTTGGGACTTAGCCAGAATAATCAAGTCGTTACACGACACTAAGTGATAAACTCAAGGTCCCTAAATTAATAcctgtctcttatacacatctccgag                                                                                                                                                      >  1:20817/1‑277 (MQ=255)`  
                                                                                                                                                     `cATGAGACGCTTTGGGACTGCATTAAGTCCATTGGCGCGAAGGCTGGTATGACCGAAGAGGATATTATCAAGCAGGGCCAAATGGCTCGAATCCTACGGTTCAACGAGTACAACTTTATTGACAAGGAGATTTACCTGTGGAGACCGCAACGTATATTGGTCTGGGTCTTTGTGTTCTCGGA‑‑GTGTGCCTCATTTCGTGGGGCCTTTGGGACTTAGCCAGAATAATCAAGTCGTTACACGACACTAAGTGATAAACTCAAGGTCCCTAAATTCATACAACTCACTATAGGGAGATAgtggg                                                                                                                                                      >  1:649747/1‑300 (MQ=255)`  
                                                                                                                                                          `gaCGCTTTGGGACTGCATTAAGGCCATTGGCGCGAAGGCTGGTATGACCGAAGAGGATATTATCAAGCAGGGCCAAATGGCTCGAATCCTACGGTTCAACGAGTACAACTTTATTGACAAGGAGATTTACCTGTGGAGACCGTAACGTATATTGGTCTGGGTCTTTGTGTTCTCGGA‑‑GTGTGCCTCATTTCGTGGGGCCTTTGGTACTTAGCCAGAATAATCAAGTCGTTACACGACACTAAGTGATAAACTCAAGGTCCCTAAATTAATACGACTCACTATAGGGAGATAGGGGCCTTTa                                                                                                                                                 <  1:213612/301‑1 (MQ=255)`  
                                                                                                                                                              `cTTTGGGACTGCATTAAGTCCATTGGCGCGAAGGCTGGTATGACCGAAGAGGATATTATCAAGCAGGGCCAAATGGCTCGAATCCTACGGTTCAACGAGTACAACTTTATTGACAAGGAGATTTACCTGTGGAGACCGTAACGTATATTGGTCTGGGTCTTTGTGTTCTCGGA‑‑GTGTGCCTCATTTCGTGGGGCCTTTGGGACTTAGCCAGAATAATCAAGTCGTTACACGACACTAAGTGATAAACTCAAGGTCCCTAAATTAATACGACTCACTATAGGGAGATAgctgtctcttataa                                                                                                                                             >  1:173716/1‑288 (MQ=255)`  
                                                                                                                                                                     `aCTGAATTAAGTCCATTGGCGCGAAGGCTGGTATGACCGAAGAGGATATTATCAAGCAGGGCCAAATGGCTCGAATCCTACGGTTCAACGAGTACAACTTTATTGACAAGGAGATTTACCTGTGGAGACCGTAACGTATATTGGTCTGGGTCTTTGTGTTCTCGGA‑‑GTGTGCCTCATTTCGTGGGGCCTTTGGGACTTAGCCAGAATAATCAAGTCGTTACACGACACTAAGTGATAAACTCAAGGTCCCTAAATTAATACGACTCACTATAGGGAGATAGGGGCCTTTACGATTATTACt                                                                                                                                      <  1:569574/301‑1 (MQ=255)`  
                                                                                                                                                                        `gCATTAAGTCCATTGGCGCGAAGGCTGGTATGACCGAAGAGGATATTATCAAGCAGGGCCAAATGGCTCGAATCCTACGGTTCAACGAGTACAACTTTATTTACAAGGAGATTTACCTGTGGAGACCGTAACGTATATTGGTCTGGGTCTTTGTGTTCTCGGA‑‑GTGTGCCTCATTTCGTGGGGCCTTTGGGACTTAGCCAGAATAATCAAGTCGTTACACGACACTAAGTGATAAACTCAAGGTCCCTAAATTAATACGACTCACTATAGGGAGATAGGGGCCTTTACGATTATTACtttt                                                                                                                                   >  1:86132/1‑300 (MQ=255)`  
                                                                                                                                                                          `aTTAAGTCCATTGGCGCGAAGGCTGGTATGACCGAAGAGGATATTATCAAGCAGGGCCAAATGGCTCGAATCCTACGGTTCAACGAGTACAACTTTATTGACAAGGAGATTTACCTGTGGAGACCGTAACGTATATTGGTCTGGGTCTTTGTGTTCTCGGA‑‑GTGTGCCTCATTTCGTGGGGCCTTTGGGACTTAGCCAGAATAATCAAGTCGTTACACGACACTAAGTGATAAACTCAAGGTCCCTAAATTAATACGACTCACTCTAGGGAGATAGGGGCCTTTACGATTATTACttctgt                                                                                                                                 >  1:360530/1‑297 (MQ=255)`  
                                                                                                                                                                                 `ccATTGGCGCGAAGGCTGGTATGACCGAAGAGGATATTATCAAGCAGGGCCAAATGGCTCGAATCCTACGGTTCAACGAGTACAACTTTATTGACAAGGAGATTTACCTGTGGAGACCGTAACGTATATTGGTCTGGGTCTTTGTGTTCTCGGA‑‑GTGTGCCTCATTTCGTGGGGCCTTTGGGACTTAGCCAGAATAATCAAGTCGTTACACGACACTAAGTGATAAACTAAAGGTCCCTAAATTAATACGACTCACTATAGGGAGATAGGGGCCTTTACGATTATTACTTTAAgctgtctt                                                                                                                          >  1:670036/1‑294 (MQ=255)`  
                                                                                                                                                                                      `ggCGCGAAGGCTGGTATGACCGAAGAGGATATTATCAAGCAGGGCCAAATGGCTCGAATCCTACGGTTCAACGAGTAAAACTTTATTGACAAGGAGATTTACCTGTGGAGACCGTAACGTATATTGGTCTGGGTCTTTGTGTTCTCGGA‑‑GTGTGCCTAATTTCGTGGGGCCTTTAGGACTTAGCCAGCATAATCAAGTCGTTACAGTACACTAAGTGATAAACTCAAGGGCGCAAAATTAATACGACTCACAATAGGGAGATAGGGGGCTTTACGATGATTACTTTAAGATTTAACTCTaa                                                                                                                     >  1:378571/1‑301 (MQ=255)`  
                                                                                                                                                                                              `ggCTGGTATGACCGAAGAGGATATTATCAAGCAGGGCCAAATGGCTCGAATCCTACGGTTCAACGAGTACAACTTTATTGACAAGGAGATTTACCTGTGGAGACCGTAACGTATATTGGTCTGGGTCTTTGTGTTCTCGGA‑‑GTGTGCCTCATTTCGTGGGGCCTTTGGGACTTAGCCAGAATAATCAAGTCGTTACACGACACTAAGTGATAAACTCAAGGTCCCTAAATTAATACGACTCACTATAGGGAGATAGGGGCCTTTACGATTATTACTTTAAGATTTAACTCTAAGAGGAAtt                                                                                                             >  1:404843/1‑300 (MQ=255)`  
                                                                                                                                                                                              `aaagagacagACCGAAGAGGATATTATCAAGCAGGGCCAAATGGCTCGAATCCTACGGTTCAACGAGTACAACTTTATTGACAAGGAGATTTACCTGTGGAGACCGTAACGTATATTGGTCTGGGTCTTTGTGTTCTCGGA‑‑GTGTGCCTCATTTCGTGGGGCCTTTGGGACTTAGCCAGAATAATCAAGTCGTTACACGACACTAAGTGATAAACTCAAGGTCCCTAAATTAATACGACTCACTATAGGGAGATAGGGGCCTTTACGATTATTACTTTAAGATTTAACTCTAAGAGGAATc                                                                                                             <  1:29663/292‑1 (MQ=255)`  
                                                                                                                                                                                               `gCTGGTATGACCGAAGAGGATATTATCAAGCAGGGCCAAATGGCTCGAATCCTACGGTTCAACGAGTACATCTTTATTGACAAGGAGATTTACCTGTGGAGACCGTAACGTATATTGGTCTGGGTCTTTGTTTTCTCGGA‑‑GTGTGCCTCATTTCGTGGGGCCTTTGGGACTTAGCCAGAATAATCAAGTCGTTACACGACACTAAGTGATAAACTAAAGGTCCCTAAATTAATACGACTCACTATAGGGAGATAGGGGCCTTTACGATTATTACTTTAAGATTTAACTCTAAGAGGAATCt                                                                                                            <  1:315340/301‑1 (MQ=255)`  
                                                                                                                                                                                                 `tGGTATGACCGAAGAGGATATTATCAAGCAGGGCCAAATGGCTCGAATCCTACGGTTCAACGAGTACAACTTTATTGACAAGGAGATTTACCTGTGGAGACCGTAACGTATATTGGTCTGGGTCTTTGTGTTCTCGGA‑‑GTGTGCCTCATTTCGTGGGGCCTTTGGGACTTAGCCAGAATAATCAAGTCGTTACACGACACTAAGTGATAAACTCACGGTCCCTAAATTAATACGACTCACTATAGGGAGATAGGGGCCTTTACGATTATTACTTTAAGATTTAACTCTAAGAGGAATCttt                                                                                                          >  1:304576/1‑301 (MQ=255)`  
                                                                                                                                                                                                  `ggTATGACCGAAGAGGATATTATCAAGCAGGGCCAAATGGCTCGAATCCTACGGTTCAACGAGTACAACTTTATTGACAAGGAGATTTACCTGTGGAGACCGTAACGTATATTGGTCTGGGTCTTTGTGTTCTCGGA‑‑GTGTGCCTCATTTCGTGGGGACTTTGGGACTTAGCCAGAATATTCCAGTCGTTACACGACACTAAGTGATAAACTCTATGTCCCTAAATTAATCCGACACACTATTGGGAGATAGGGGCCTTTACGATTTGTCCTTTAAGATTTTACTCTAAGAGGAATCtttt                                                                                                         >  1:184539/1‑300 (MQ=255)`  
                                                                                                                                                                                                   `gTATGACCGAAGAGGATATTATCAAGCAGGGCCAAATGGCTCGAATCCTACGGTTCAACGAGTACAACTTTATTGACAAGGAGATTTACCTGTGGAGACCGTAACGTATATTGGTCTGGGTCTTTGTGTTCTCGGA‑‑GTGTGCCTCATTTCGTGGGGCCTTTGGGACTTAGCCAGAATAATCAAGTCGTTACACGACACTAAGTGATAAACTCAAGGTCCCTAAATTAATACGACTCACTATAGGGAGATAGGGGTCTTTACGATTATTACTTTAAGATTTAACTCTAAGAGGAATCTttat                                                                                                        >  1:515451/1‑301 (MQ=255)`  
                                                                                                                                                                                                       `gACCGAAGAGGATATTATCAAGCAGGGCCAAATGGCTCGAATCCTACGGTTCAACGAGTACAACTTTATTGACAAGGAGATTTACCTGTGGAGACCGTAACGTATATTGGTCTGGGTCTTTGTGTTCTCGGA‑‑GTGTGCCTCATTTCGTGGGGCCTTTGGGACTTAGCCAGAATAATCAAGTCGTTACACGACACTAAGTGATAAACTCACGGTCCCTAAATTAACACGACTCACTATAGGGAGATAGGGGCCTTTACGACTATTACTTTAAGATTTAACTCTAAGAGGAAtttccgtccct                                                                                                    >  1:539901/1‑291 (MQ=255)`  
                                                                                                                                                                                                        `aCCGAAGAGGATATTATCAAGCAGGGCCAAATGGCTCGAATCCTACGGTTCAACGAGTACAACTTTATTGACAAGGAGATTTACCTGTGGAGACCGTAACGTATATTGGTCTGGGTCTTTGTGTTCTCGGA‑‑GTGTGCCTCATTTCGTGGGGCCTTTGGGACTTAGCCAGAATAATCAAGTCGTTACACGACACTAAGTGATAAACTCAAGGTCCCTAAATTAATACGACTCACTATAGGGAGATAGGGGCCTTTACGATTATTACTTTAAGATTTAACTCTAAGAGGAATCTTTATTATGt                                                                                                   >  1:284453/1‑301 (MQ=255)`  
                                                                                                                                                                                                             `aagggATAGTATCAAGCAGGGCCAAATGGCTCGAATCCTACGGTTCAACGAGTACAACTTGATTGACAAGGAGATTTACCTGTGGATACCGTAACGTATATTGGTCTGGGTCTTTGTGTTCTCGGA‑‑GTGTGCCTCATTTCGTGGGGCCTTTGGGACTTAGCCAGAATAATCAAGTCGTTACACGACACTAAGTGATAAACTCAAGGTCCCTAAATTAATACGACTCACTATAGGGAGATAGGGGCCTTTACGATTATTACTTTAAGATTTAACTCTAAGAGGAATCTTTATTATGTTAaca                                                                                              <  1:557801/298‑1 (MQ=255)`  
                                                                                                                                                                                                               `ataagagacagaaGCAGGGCCAAATGGCTCGAATCCTACGGTTCAACGAGTACAACTTTATTGACAAGGAGATTTACCTGTTGAGACCGTAACGTATATTGGTCTGGGTCTTTGTGTTCTCGGA‑‑GTATGCCTCATTTCGTGGTGCCTTTGGTACTTAGCCAGAATAATCAAGTCGTTACACGACACTAAGTGATAAACTCAAGGTCCCTAAATTAATACGACTCACTATAGGGAGATAGGGGCCTTTACGATTATTACTTTAAGATTTAACTCTAAGAGGAATCTTTATTATGTTAACAcc                                                                                            <  1:170110/290‑1 (MQ=255)`  
                                                                                                                                                                                                                `ggATATTATCAAGCAGGGCCAAATGGCTCGAATCCTACGGTTCAACGAGTACAACTTTATTGACAAGGAGATTTACCTGTGGAGACCGTAACGTATATTGGTCTGGGTCTTTGTGTTCTCGGA‑‑GTGTGCCTCATTTCGTGGGGCCTTTGGGACTTAGCCAGAATCATCAAGTCGTTACACGACACTAAGTGATAAACTCAAGGTCCCTAAATTAATACGACTCACTATAGGGAGATAGGGGCCTTTACGATTATTACTTTAAGATTTAACTCTAAGAGGAATCTTTATTATGTTAACACCt                                                                                           >  1:202024/1‑301 (MQ=255)`  
                                                                                                                                                                                                                `ggATATTATCAAGCAGGGCCAAATGGCTCGAATCCTACGGTTCAACGAGTACAACTTTATTGACAAGGAGATTTACCTGTGGAGACCGTAACGTATATTGGTCTGGGTCTTTGTGTTCTCGGA‑‑GTGTGCCTCATTTCGTGGGGCCTTTGGGACTTAGCCAGAATAATCAAGTCGTTACACGACACTAAGTGATAAACTCAAGGTCCCTAAATTAATACGACTCACTATAGGGAGATAGGGGCCTTTACGATTATTACTTTAAGATTTAACTCTAAGAGGAATCTTTATTATGTTAACACCt                                                                                           >  1:265652/1‑301 (MQ=255)`  
                                                                                                                                                                                                                 `gATATTATCAAGCAGGGCCAAATGGCTCGAATCCTACGGTTCAACGAGTACAACTTTATTGACAAGGAGATTTACCTGTGGAGACCGTAACGTATATTGGTCTGGGTCTTTGTGTTCTCGGA‑‑GTGTGCCTCATTTCGTGGGGCCTTTGGGACTTAGCCAGAATAATCAAGTCGTTACACGACACTAAGTGATAAACTCAAGGTCCCTAAATTAATACGACTCACTATAGGGAGATAGGGGCCTTTACGATTATTACTTTAAGATTTAACTCTAAGAGGAATCTTTATTATGTTAACACCtt                                                                                          >  1:451362/1‑300 (MQ=255)`  
                                                                                                                                                                                                                  `atatTATCAAGCAGGGCCAAATGGCTCGAATCCTACGGTTCAACGAGTACAACTTTATTGACAAGGAGATTTACCTGTGGAGACCGTAACGTATATTGGTCTGGGTCTTTGTGTTCTCGGA‑‑GTGTGCCTCATTTCGTGGGGCCTTTGGGACTTAGCCAGAATAATCAAGTCGTTACACGACACTAAGTGATAAACTCAAGGTCCCTAAATTAATACGACTCACTATAGGGAGATAGGGGCCTTTACGATTATTCCTTTAAGATTTAACTCTAAGAAGAATCTTTATTATGTTAACACCTAt                                                                                         >  1:416579/1‑301 (MQ=255)`  
                                                                                                                                                                                                                     `ttatCAAGCAGGGCCAAATGGCTCGAATCCTACGGTTCAACGAGTACAACTTTATTGACAAGGAGATTTACCTGTGGAGACCGTAACGTATATTGGTCTGGGTCTTTGTGTTCTCGGA‑‑GTGTGCCTCATTTCGTGGGGCCTTTGGGACTTAGCCAGAATAATCAAGTCGTTACACGACACTAAGTGATAAACTCAAGGTCCCTCAATTAATATGACTCACTATAGGGAGATAGGGGCCTTTACGATTATTACTTTAAGATTTAACTCTAAGAGGAATCTTTATTATGTTAACACCTATTaa                                                                                      >  1:509447/1‑301 (MQ=255)`  
                                                                                                                                                                                                                     `ttatCAAGCAGGGCCAAATGGCTCGAATCCTACGGTTCAACGAGTACAACTTTATTGACAAGGAGATTTACCTGTGGAGACCGTAACGTATATTGGTCTGGGTCTTTGTGTTCTCGGA‑‑GTGTGCCTCATTTCGTGGGGCCTTTGGGACTTAGCCAGAATAATCAAGTCGTTACACGACACTAAGTGATAAACTCAAGGTCCCTAAATTAATACGACTTACTATAGGGAGATAGGGGCCTTTACGCTTATTACTTTAAGATTTAACTCTAAGAGGAATCTTTATTATGTTAACACCTATTaa                                                                                      >  1:246634/1‑301 (MQ=255)`  
                                                                                                                                                                                                                     `ttatCAAGCAGGGCCAAATGGCTCGAATCCTACGGTTCAACGAGTACAACTTTATTGACAAGGAGATTTACCTGTGGAGACCGTAACGTATATTGGTCTGGGTCTTTGTGTTCTCGGA‑‑GTGTGCCTCATTTCGTGGGGCCTTTGGGACTTAGCCAGAATAATCAAGTCGTTACACGACACTAAGTGATAAACTCAAGGTCCCTAAATTAATACGACTCACTATAGGGAGATAGGGGCCTTTACGATTATTACTTTAATATTTAACTCTAAGAGGAATCTTTATTATgctgtctcttataca                                                                                      >  1:165006/1‑287 (MQ=255)`  
                                                                                                                                                                                                                     `ttatCAAGCAGGGCCAAATGGCTCGAATCCTACGGTTCAACGAGTACAACTTTATTGACAAGGAGATTTACCTGTGGAGACCGTAACGTATATTGGTCTGGGTCTTTGTGTTCTCGGA‑‑GTGTGCCTCATTTCGTGGGGCCTTTGGGACTTAGCCAGAATAATCAAGTCGTTACACGACACTAAGTGATAAACTCAAGGTCCCTAAATTAATACGACTCACTATAGGGAGATAGGGGCCTTTACGATTATTACTTTAAGATTTAACTCTAAGAGGAATCTTTATTATGTTAACACCTATTaa                                                                                      >  1:644064/1‑301 (MQ=255)`  
                                                                                                                                                                                                                      `tatCAAGCAGGGCCAAATGGCTCGAATCCTACGGTTCAACGAGTACAACTATATTGACAAGGAGATTTACCTGTGGAGACCATAACGTATATTGGTCTGGGTCTTTGTGTTCTCGGA‑‑GTTTGCCTCATTTCGTGGGGCCTTTGGGACTTAGCCAGAATAATCAAGTCGTTACACGACACTAAGTGATAAACTCAAGGTCCCTAAATTAATACGACTCACTATAGGGAGATAGGGGCCTTTACGATTATTACTTTAAGATTTAACTCTAAGAGGAATCTTTATTATGTTAACACCTATTAAc                                                                                     <  1:332473/301‑1 (MQ=255)`  
                                                                                                                                                                                                                       `ttCAAGAAGGGTCAAATGGTCCGAAGCCGACGGTTCAACGAGTACAACGTTATTGAAAAGGAGAGTTAACTGTGGAGACAGTAACGTATATTGGTATGGGTCTTTGTGTTCTCGGA‑‑GTGTGGCTCATTTCGTGGGGCCTTTGGGACTTAGCCAGAATAATCAAGTCGTTACACGACACTAAGTGATAAACTCAAGGTCCCTAAAGTAATACGACTCACTATAGGGAGATAGGGGCCTTTACGATTATTACTTTAAGATTTAACTCTAAGAGGAATCTTTATTATGTTAACACCTATTAAcc                                                                                    <  1:623327/300‑1 (MQ=255)`  
                                                                                                                                                                                                                       `atCAAGCAGGGCCAAATGTCTCGAGTCCTACGGTTGAACGAGTACAACTTTATTGACAAGTAGATTTACCTGTGGAGACCGTAACTTATATTGGTCTGGGTCTTTGTGTTCTCGGA‑‑GTGTGCCTCATTTCGTGGGGCCTTTGGGACTTAGCCAGAATAATCAATTCGTTATACGACACTAAGTGATAAACTCAAGGTCCCTAAATTAATACGACTCACTATAGGGAGATAGGGGCCTTTACGATTATTACTTTAAGATTTAACTCTAAGAGGAATCTTTATTATGTTAACACCTATTAAcc                                                                                    <  1:355084/301‑1 (MQ=255)`  
                                                                                                                                                                                                                       `atCAAGCAGGGCCAAATGGCTCGAATCCTACGGTTCAACGAGTACAACTTTATTGACAAGGAGATTTACCTGTGGAGACCGTAACGTATATTGGTCTGGGTCTTTGTGTTCTCGGA‑‑GTGTGCCTCATTTCGTGGGGCCTTTGGGACTTAGCCAGAATAATCAAGTCGTTACACGACACTAAGTGATAAACTCAAGGTCCCTAAATTAATACGACTCACTATAGGGAGATAGGGGCCTTTACTATTATTACTTTAAGATTTAACTTTAAGAGGAATCTTTATTATTTTAACACCTATTAAcc                                                                                    >  1:366656/1‑301 (MQ=255)`  
                                                                                                                                                                                                                                         `gCTCGAATCCTACGGTTCAACGAGTACAACTTTATTGACAAGGAGATTTACCTGTGGAGACCGTAACGTATATTGGTCTGGGTCTTTGTGTTCTCGGA‑‑GTGTGCCTCATTTCGTGGGGCCTTTGGGACTTCGCCAGAATAATCAAGTCGTTACACGACACTAAGTGATACACTCCAGGTCCCTAAATTACTACGACTCACTATAGGGAGATAGGGGCCTTTACGATTTTTACTTTAAGATTTAACTCTAATAGGAATCTTTATTCTGTTAACACCTATTAACCAATTACTTAAGAACCCtt                                                                  >  1:502710/1‑300 (MQ=255)`  
                                                                                                                                                                                                                                             `gAATCATACGTGTCAAGGAGTATAACTTTATTGAGAAGGAGATTTACCGTTGGAGACAGTAACGTTTATTGTTCTGATTTTTGATGTTATAGGA‑‑GTGTGCCTCATTTCGTGGGGACTTTGGGACTTAGCCAGAATAATCAAATCGGTACACGACACTAAGTGATAAACTCAAGGTCCCTAAAGTAATACGACTCACTATAGGGAGATATGGGCCTTTACGATTATTACTTTAAGATTTAACTCTAAGAGAAATCTTTATTATGTTAACACCTATTAACCAATTACTTAAGAACCCTAACGa                                                              <  1:397755/301‑1 (MQ=255)`  
                                                                                                                                                                                                                                               `aTCCTATGGTTCAACGAGTACAACTTTATTGACAAGGAGATTTACCTGTGGAGACCGTAACGTATATTGGTCTGGGTCTTTGTGTTCTCGGA‑‑GTGTGCCTCATTTCGTGGGGCCTTTGGGACTTAGCCAGAATAATCAAGTCGTTACACGACACTAAGTGATAAACTCAAGGTCCCTAAATTAATACGACTCACTATAGGGAGATAGGGGCCTTTACGATTATTACTTTAAGATTTAACTCTAAGAGGAATCTTTATTATGTTAACACCTATTAACCAATTACTTAAGAACCCTAACGatt                                                            >  1:196478/1‑300 (MQ=255)`  
                                                                                                                                                                                                                                                  `cTACGGTTCAACGAGTACAACTTTATTGACAAGGAGATTTACCTGTGGAGACCGTAACGTATATTGGTCTGGGTCTTTGTGTTCTCGGA‑‑GTGTGCCTCATTTCGTGGGGCCTTTGGGACTTAGCCAGAATAATCAAGTCGTTACACGACACTAAGTGATAAACTCAAGGTCCCTAAATTAATACGACTCACTATAGGGAGATAGGGGCCTTTACGATTATTACTTTAAGATTTAACTCTAAGAGGAATCTTTATTATGTTAACACCTATTAACCAATTACTTAAGAAcctgtatcttataa                                                         >  1:107740/1‑289 (MQ=255)`  
                                                                                                                                                                                                                                                     `cGGTTCAACGAGTACAACTTTATTTACACGGAGATTTACCTGTGGAGACCGTAACGTATATTGGTCTGGGTCTTTGTGTTCTCGGA‑‑GTGTGCCTCATTTCGTGGGGCCTTTGGGACTTATCCAGAATAATCAAGTCGTTTCACGACACTAAGTGATAAACTCAAGGTCCCTAAATTAATACGACTCACTATAGGGAGATAGGGGCCTTTACGATTATTACTTTAAGATTTAACTCTAAGAGGAATCTTTATTATGTTAACACCTATTAACCAATTACTTAAGAACCCTAACGATATTCCAg                                                      <  1:655674/301‑1 (MQ=255)`  
                                                                                                                                                                                                                                                      `ggTTCAACGAGTACAACTTTATTGACAAGGAGAGTTACCTGTGGAGACCGTAACGTATATGGGTCTGGGTCTTTGTGTTCTCGGA‑‑GTGTGCCTCATTTCGTGGGGCCTTTGGGACTTAGCCAGAATAATCAAGTCGTTACACGACACTAAGTGATAAACTCAAGGTCCCTAAATTAATACGACTCACTATAGGGAGATAGGGGCCTTTACGATTATTACTTTAAGATTTAACTCTAAGAGGAATCTTTATTATGTTAACACCTATTAACCAATTACTTAAGAACCCTAACGATATTCCAGa                                                     <  1:39170/301‑1 (MQ=255)`  
                                                                                                                                                                                                                                                       `gTTCAACGAGTACAACTTTATTGACAAGGAGATTTACCTGTGGAGACCGTAACGTATATTGGTCTGGGTCTTTGTGTTCTCGGA‑‑GTGTGCCTCATTTCGTGGGGCCTTTGGGACTTAGCCAGAATAATCAAGTCGTTACACGACACTAAGTGATAAACTCAAGGTCCCTAAATTAATACGACTCACTATAGGGAGATAGGGGCCTTTACGATTATTACTTTAAGATTTAACTCTAAGAGGAATCTTTATTATGTTAACACCTATTAACCAATTACTTAAGAACCCTAACGATATTCCAGAt                                                    >  1:179545/1‑301 (MQ=255)`  
                                                                                                                                                                                                                                                                      `cTTTATTGACAAGGAGATTTACCTGTGGAGACCGTAACGTATATTGGTCTGGGTCTTTGTGTTCTCGGA‑‑GTGTGCCTCATTTCGTGGGGCCTTTGGGACTTAGCCAGAATAATCAAGTCGTTACACGACACTAAGTGATAAGCTCAAGGTCCCTAAATTAATACGACTCACTATAGGGAGATAGGGGCCTTTACGATTATTACTTTAAGATTTAACTCTAAGAGGAATCTTTATTATGTTAACACCTATTAACCAATTACTTAAGAACCCTAACGATATTCCAGATgctgtctcttataca                                     >  1:25856/1‑287 (MQ=255)`  
                                                                                                                                                                                                                                                                        `ttATTGACAAGGAGATTTACCTGTGGAGACCGTAACGTATAGTGGTCTGGGTCTTTGTGTTCTCGGA‑‑GTGTGCCTCATTTCGTGGTGCCTTTGGGACTTAGCCAGAATAATCAAGTCGTTACACGACACTAAGTGATAAACTCAAGGTCCCTAAATTAATACGACTCACTATAGGGAGATAGGGGCCTTTACGATTATTACTTTAAGATTTAACTCTAAGAGTAATCTTTATTATGTTAACACCTATTAACCAATTACTTAAGAACCCTAACGATATTCCAGATGTACCTCGTGCAACCGc                                   <  1:190748/301‑1 (MQ=255)`  
                                                                                                                                                                                                                                                                             `gACAAGGAGATTTACCTGTGGAGACCGTAACGTATATTGGTCTGGGTCTTTGTGTTCTCGGA‑‑GTGTGCCTCATTTCGTGGGGCCTTTGGGACTTAGCCAGAATAATCAAGTCGTTACACGACACTAAGTGATAAACTCAAGGTCCCTAAATTAATACGACTAACTATAGGGAGATAGGGGCCTTTACGATTATTACTTTAAGATTTAACTCTAAGAGGAATCTTTATTATGTTAACACCTATTAACCAATTACTTAAGAACCATAACGATATTCCAGATGTACCTCGTGCAACCGcctgtc                              >  1:100050/1‑296 (MQ=255)`  
                                                                                                                                                                                                                                                                               `cAAGGAGATTTACCTGTGGAGACCGTAACGGATATTGGGCTGGGTCTTTGTGTTCGCGGA‑‑GTGTGCCGCATTTTGTGGGGCCTTTGGGACTTAGCCAGAATAATAAAGTCGTTACACGACACTAAGTGATAAACTAAAGGTCGATAAATTAATACGACTCACTATAGGGAGATAGGGGCCTTTACGATTATTAATTTAAGATTTAACTCTAAGCGGACTCTTTATTATGATAACAAGTATTAACCAATTGCTTAAGAACGCTAACGATATTCAAGATGTActcagttcagccgttgagaaa                            >  1:598052/1‑281 (MQ=255)`  
                                                                                                                                                                                                                                                                               `aaaGGAGATTTATCTGTGGAGACCGTAACGTATATTGGTCTGGGTCTTTGTGTTCTCGGA‑‑GTGTGCCTCATTTCGTGGGGCCTTTGGGACTTAGCCAGAATAATCAAGTCGTTACAAGACACTAAGTGATAAACTCAAGGTCCCTAAATTAATACGACTCACTATAGGGAGATAGGGGCCTTTACGATTATTACTTTAAGATTTAACTCTAAGAGGAATCTTTATTATGTTAACACCTATTAACCAATTACTTAAGAACCCTAACGATATTCCAGATGTACCTCGTGCAACCGCTGAGTAt                            <  1:636362/300‑1 (MQ=255)`  
                                                                                                                                                                                                                                                                                    `agaTTTACCTGTGGAGACCGTGACGTATATTGGTCTGGGTCTTTGTGTTCTCGGA‑‑GTGTGCCTCATTTCGTGGGGCCTTTGGGACTTAGCCAGAATAATCAAGTCGTTACACGACACTAAGTGATAAACTCAAGGTCCCTAAATTAATACGACTCACTATAGGGAGATAGGGGCCTTTACGATTATTACTTTAAGATTTAACTCTAAGAGGAATCTTTATTATGTTAACACCTATTAACCAATTACTTAAGAACCCTAACGATATTCCAGATGTACCTCGTGCAACCGCTGAGTATCTACa                       <  1:422928/301‑1 (MQ=255)`  
                                                                                                                                                                                                                                                                                      `agagacaggtgGAGACCTTAACGTATATTGGTCTGGGTCTTTGTGTTCTCGGA‑‑GTGTGCCTCATTTCGTGGGGCCTTTGGGACTTAGCCAGAATAATCAAGTCGTTACACGACACTAAGTGATAAACTCAAGGTCCCTAAATTAATACGACTCACTATAGGGAGATAGGGGCCTTTACGATTATTACTTTAAGATTTAACTCTAAGAGGAATCTTTATTATGTTAACACCTATTAACCAATTACTTAAGAACCCTAACGATATTCCAGATGTACCTCGTGCAACCGCTGAGTATCTACAgg                     <  1:76090/293‑1 (MQ=255)`  
                                                                                                                                                                                                                                                                                              `gtgGAGACCGTAACGTATATTGGTCTGGGTCTTTGTGTTCTCGGA‑‑GTGTGCCTCATTTCGTGGGGCCTTTGGGACTTAGCCAGAATAATCAAGTCGTTACACGACACTAAGTGCTCAACTAAAGGTCCCTAAATTAATACGACTTACTATAGGGAGATAGCGGCCCTTACGATTATTACTTTAAGCTTTAACTCTAAGAAGAATCTTTATTCTGTTATCATATATTAAAAAATTACTTAAGAACCCTAACGATATTCCAGATTTACCTCGTCTCACCGCTGACTATATACACGTTCGCTTc             >  1:439943/1‑301 (MQ=255)`  
                                                                                                                                                                                                                                                                                              `gtgGAGACCGTAACGTATATTGGTCTGGGTCTTTGTGTTCTCGGA‑‑GTGTGCCTCATTTCGTGGGGCCTTTGGGACTTAGCCAGAATAATCAAGTCGTTACACGACACTAAGTGATAAACTCAAGGTCCCTAAATTAATACGACTCACTATAGGGAGATAGGGGCCTTTACGATTATCACTTTAAGATTTAACTCTAAGAGGAATCTTTATTATGTTAACACCTATTAACCAATTACTTAAGAACCCTAACGATATTCCAGATGTACCTCGTGCAACCGCTGAGTATCTACAGGTTCGAtta             >  1:385270/1‑300 (MQ=255)`  
                                                                                                                                                                                                                                                                                                `ggAGACCGTAACGTATATTGGTCTGGGTCTTTGTGTTCTCGGA‑‑GTGTGCCTCATTTCGTGGGGCCTTTGGGACTTAGCCAGAATAATCAAGTCGTTACACGACACTAAGTGATAAACTCAAGGTCCCTAAATTAATACGACTCACTATAGGGAGATAGGGGCCTTTACGATTATTACTTTAAGATTTAACTCTAAGAGGAATCTTTATTATGTTAACACCTATTAACCAATTACTTAAGAACCCTAACGATATTCCAGATGTACCTCGTGCAACCGCTGAGTATCTACAGGTTCGATTaaa           >  1:290062/1‑301 (MQ=255)`  
                                                                                                                                                                                                                                                                                                 `gagaCCGTAACGTAGATTGGTCTGGGTCTTTGTGTTCTCGGA‑‑GTGTGCCTCATTTCGTGGGGCCTTTGGGACTTAGCCAGAATAATCAAGTCGTTACACGACACTAAGTGATAAACTCAAGGTCCCTAAATTAATACGACTCACTATAGGGAGATAGGGGCCTTTACGATTATTACTTTAAGATTTAACTCTAAGAGGAATCTTTATTATGTTAACACCTATTAACCAATTACTTAAGAACCCTAACGATATTCCAGATGTACCTCGTGCAACCGCTGAGTATCTACAGGTTCGATTCAAc          <  1:460837/301‑1 (MQ=255)`  
                                                                                                                                                                                                                                                                                                   `gaCTGTAACGTATATTGTTCTGGTTCTTTGCGGTCTCGGA‑‑GTGTGCCTCATTTCGTGTTGCCTTTTGGACTTAGCCAGAATAACCAAGTCATAACACGACACTAAGTGATATACTCAAGTTCCCTAAATTAATATAACTCACTATAGGGATATAGTTGCCTTGATGATTATTACTTTAAGATTTAACTCTAAGATGAATCTTTATTATGTTAACACCTATTAAACAATTACTTAAGAACCCTAACGATATTCCAGATGTACCTCGTGCAACCGCTGAGTATCTACAGGTTCGATTCAACTa        <  1:337516/301‑1 (MQ=255)`  
                                                                                                                                                                                                                                                                                                   `gaCCGTAACGTATATTGGTCTGGGTCTTTGGGTTCTCGGA‑‑GTGTGCCTCATTTCGTGGGGCCTTTGGGACTTAGCCAGAATAATCAAGTCGTTACACGACACTAAGTGATAAACTCAAGGTCCCTAAATTAATACGACTCACTATAGGGAGATAGGGGCCTTTACGATTATTACTTTAAGATTTAACTCTAAGAGGAATCTTTATTATGTTAACACCTATTAACCAATTACTTAAGAACCCTAACGATATTCCAGATGTACCTCGTGCAACCGCTGAGTATCTACAGGTTCGATTCAACTa        <  1:551936/301‑1 (MQ=255)`  
                                                                                                                                                                                                                                                                                                   `gaCCGTAACGTATATTGGACTGGGTCTTTGTGTTCTCGGA‑‑GTGTGCCTCATTTCGTGGGGCCTTTGGGACTTAGCCAGAATAATCAAGTCGTTACACGACACTAAGTGATAAACTCAAGGTCCCTAAATTAATACGACTCACTATAGGGAGATAGGGGCCTTTACGATTATTACTTTAAGATTTAACTCTAAGAGGAATCTTTATTATGTTAACACCTATTAACCCATTACTTAAGAACCCTACCAATATTCCAGATGTACCTCGTGCAAACGCTGAGTATCTACAGGTTCGCTTACACTa        >  1:605077/1‑301 (MQ=255)`  
                                                                                                                                                                                                                                                                                                   `agagacaGCGTATATTGGTCTGGGTCTTTGTGTTCTCGGA‑‑GTGTGCCTCATTTCGTGGGGCCTTTGGGACTTAGCCAGAATAATCAAGTCGTTACACGACACTAAGTGATAAACTCAAGGTCCCTAAATTAATACGACTCACTATAGGGAGATAGGGGCCTTTACGATTATTACTTTAAGATTTAACTCTAAGAGGAATCTTTATTATGTTAACACCTATTAACCAATTACTTAAGAACCCTAACGATATTCCAGATGTACCTCGTGCAACCGCTGAGTATCTACAGGTTCGATTCAACTa        <  1:523635/295‑1 (MQ=255)`  

                                                                                                                                                                                                                                                                                                          `|`                                                                                                                                                                                                                                                                                                               
`ATGGTTACTCAGGGATTGCTGGATGGGGTGATACCGCCGAGGACTTCTTGAATAACCCGTTCATAACCGAGCCTAAAACGTCTGTGCTTAAGTCCGGTAAGAACAAAGGCCAAGAGGTTACTAAATGGGTTAAACGCGACCCTGAGCCTCATGAGACGCTTTGGGACTGCATTAAGTCCATTGGCGCGAAGGCTGGTATGACCGAAGAGGATATTATCAAGCAGGGCCAAATGGCTCGAATCCTACGGTTCAACGAGTACAACTTTATTGACAAGGAGATTTACCTGTGGAGACCGTAGCGTATATTGGTCTGGGTCTTTGTGTTCTCGGA‑‑GTGTGCCTCATTTCGTGGGGCCTTTGGGACTTAGCCAGAATAATCAAGTCGTTACACGACACTAAGTGATAAACTCAAGGTCCCTAAATTAATACGACTCACTATAGGGAGATAGGGGCCTTTACGATTATTACTTTAAGATTTAACTCTAAGAGGAATCTTTATTATGTTAACACCTATTAACCAATTACTTAAGAACCCTAACGATATTCCAGATGTACCTCGTGCAACCGCTGAGTATCTACAGGTTCGATTCAACTATGCGTA  >  NC_001604.1/18108‑18705`

  

Alignment Legend

Aligned base mismatch/match (shaded by quality score): `ATCG/ATCG` `< 3 ≤` `ATCG/ATCG` `< 10 ≤` `ATCG/ATCG` `< 21 ≤` `ATCG/ATCG` `< 34 ≤` `ATCG/ATCG` `< 38 ≤` `ATCG/ATCG`

Unaligned base: `atcg`    Masked matching base: `atcg`    Alignment gap: `‑`    Deleted base: `‑`
